# Maternal and neonatal exposure to risk factors for neonates with moderate or severe hypoxic ischemic encephalopathy: a cross-sectional study

**DOI:** 10.1186/s13052-022-01380-w

**Published:** 2022-11-26

**Authors:** Yiran Wang, Shuying Luo, Kaijuan Wang, Yuwei Hou, Hui Yan, Yaodong Zhang

**Affiliations:** 1 Henan Key Laboratory of Children’s Genetics and Metabolic Diseases, Children’s Hospital Affiliated to Zhengzhou University, Henan Children’s Hospital, Zhengzhou Children’s Hospital, Henan Province, Zhengzhou, China; 2 Department of Epidemiology and Health Statistics, College of Public Health, Zhengzhou University, Henan Province, Zhengzhou, China

**Keywords:** Hypoxic ischemic encephalopathy, Risk factors, Cross-sectional study

## Abstract

**Background:**

To investigate the association between maternal and neonatal exposure to the relevant influencing factors and risk of moderate or severe hypoxic ischemic encephalopathy (HIE), and the possible interactions in the Chinese population.

**Methods:**

A cross-sectional study comprising 228 neonates from Henan Children’s Hospital during the five-year period 2015–2020 in China was conducted. All neonatal basic demographic information and clinical records were documented from the neonatal HIE database. Comparisons between mild HIE and moderate or severe HIE were conducted with the t-test or Wilcoxon rank-sum test for continuous variables and the Chi-square test for categorical variables. Unconditional multiple logistic regression models were used to generate the odds ratios(ORs) and 95% confidence intervals(CIs). In addition, we also used an additive model to test for possible biological interactions among the factors.

**Results:**

Of the 228 neonates, the males had a statistically significantly higher frequency compared with the females between the two groups (*P* = 0.030). Trend analysis results found that with the decreased of the neonatal birth weight, the detection rates of moderate or severe HIE in males and females were gradually increased (*P*_trend_ < 0.05). The detection of moderate or severe HIE in males and females increased with the decreased of neonatal gestational age at birth(*P*_trend_ < 0.05). However, no interaction was detected between neonatal birth weight and gestational age at birth based on the additive model, the Relative Excess Risk of Interaction and 95% *CI* was 0.821(-0.046,1.687). The adjusted multiple logistic regression model showed that low birth weight(*OR*_*adj*_:1.965, 95%*CI*:1.086–4.127),premature infant(*OR*_*adj*_:1.557, 95%*CI*:1.589–4.862),1-min Apgar’s score < 7(*OR*_*adj*_:5.618, 95%*CI*:3.724–7.353),intrauterine distress(*OR*_*adj*_:4.916, 95%*CI*:3.431–7.398),amniotic fluid contamination (*OR*_*adj*_:3.965, 95%*CI*:2.153–5.782) significantly increased the risk of neonatal moderate or severe HIE.

**Conclusion:**

Neonates with low birth weight, premature infant,1-min Apgar’s score < 7, intrauterine distress, amniotic fluid contamination are risk factors for moderate or severe HIE. Notably, we found no biological interaction between risk factors based on the additive model, these findings may help to inform prevention strategies, as this may effectively reduce the incidence of neonatal moderate or severe HIE.

## Introduction

Neonatal Hypoxic ischemic encephalopathy (HIE) is a common disease caused by various factors in the whole perinatal period of neonates, which possibly causes neonatal hypoxia, reduced cerebral blood flow or suspension, thus resulting in varying degrees of brain damage [1,2]. Based on the neurological symptoms of the neonates, HIE is graded into three categories: mild, moderate and severe [3]. For neonates with mild HIE, outcomes are consistently positive, in a retrospective cohort study of neonates ≥ 35 weeks of gestation with mild HIE who underwent therapeutic hypothermia to correlate the early electroencephalography background pattern with clinical course and outcomes found that 19 had either a normal or a mildly abnormal background, neonates with mild HIE are more likely to survive without developmental disabilities than those with moderate or severe HIE, whereas neonates with moderate to severe HIE can suffer serious consequences, including death, cerebral palsy, epilepsy and cognitive disability [4,5]. Neonatal HIE accounts for 15.2% of mortality under the age of five, in addition, the mortality rate of neonatal moderate or severe HIE is high, and the rate of severe neurological disability in survivors is up to 55%-73% [6]. Despite of significant improvements in neonatal care, HIE continues to exist in developed countries and a disproportionately high burden remains in low and middle income countries [7–11]. There are 15–18 million live births in China each year, and the incidence of neonatal HIE is estimated to be 3–6 per 1000 live births [12].

To our knowlegde, neonatal HIE is the most common cause of seizures in newborns and accounts for approximately half of all cases. Neonatal HIE with seizures are associated with high risk of death and chronic morbidities, such as epilepsy, cerebral palsy, developmental delays and cognitive impairment. Neonatal seizures include those critical events whose onset has occurred during the first 28 days of life and till date. The incidence of neonatal seizures is estimated to be 1 to 3 per 1000 for term infants and much higher for preterm infants [13,14]. Neonatal HIE with seizures treatment is a severely importance subject in child neurology. Levetiracetam is a newer antiepileptic drug that is U.S. Food and Drug Administration approved for the treatment of epilepsy in children aged 1 month and older. Several studies [15–19] have demonstrated children exposed to levetiracetam in utero may have less risk of poor development. Furthermore, levetiracetam does not exhibit a proapoptotic effect in neurons. The use of levetiracetam independently efficient in treating neonatal seizures, while significantly decreasing the risk probability for adverse events. Improved treatments for neonatal HIE with seizures may decrease morbidity and mortality associated with the disease.

Clinically, it is urgent need to identify the relevant risk factors of the neonatal moderate or severe HIE to take appropriate preventive and early intervention strategies before neonates occur. Previous epidemiological studies have demonstrated a number of medical and socioeconomic risk factors for neonatal moderate or severe HIE [20–23], including maternal age, maternal obesity, gestational diabetes,viral infection, intrauterine growth retardation, neonatal mode of delivery, birth weight, gestational age, but these findings may somewhat inconsistent considering the changes in study designs, characteristics of the population, diagnostic criteria and outcome measures, meanwhile, several studies lacked analysis of the possible interactions among risk factors. Therefore, a cross-sectional study was undertaken in this study to identify the independent risk factors of neonatal moderate or severe HIE and also address the possible biological interactions used an additive model, in order to provide the necessary reference for early primary and secondary prevention in clinical.

## Methods

### Study population

This was a cross-sectional study comprising 228 neonates from Henan Children’s Hospital during the five-year period 2015–2020 in China. All neonatal basic demographic information and clinical records were documented from the neonatal HIE database. The neonatal HIE database was set up in Henan Children’s Hospital and known to be a province-wide, clinically-oriented computerized disease-specific database. We obtained information on the neonatal age at diagnosis, gender, residential region, birth weight and delivery mode from their birth certificate cards or vaccination cards and also collected related medical data from their medical records in the hospital. To ensure the reliability of the information, we routinely examined the items and logical errors in the database. When data was found to be inconsistent, we would list the numbers of the patients, record names, variable name and error categories to facilitate future checks and corrections.

For the current study, to focus on moderate and severe HIE, cases of HIE were defined by the following criteria:(a) all the 228 HIE cases diagnostic criteria were set by the Neonatal Group of the Pediatric Society of Chinese Medical Association; (b) all 228 neonates resided in the Neonatal Intensive Care Unit (NICU) of Henan Children’s Hospital and had typical clinical manifestations (paroxysmal cyanosis, agitation, increased muscle tone, drowsiness, full anterior fontanelle, low muscle tone, binocular gaze, abnormal emission, screaming, convulsions, etc.), which were confirmed by head CT, magnetic resonance imaging (MRI) and other examinations;(c) the parents of the neonates were able to cooperate closely with the investigation and the data were complete. According to clinical classification, neonatal HIE was divided into three categories: mild mainly manifested as agitation, normal or slightly active primitive reflex, no convulsion; moderate (mainly with inhibition symptoms, manifested as drowsiness, decreased muscle tone, weakened primitive reflex, partially with increased intracranial pressure and convulsion), severe (mainly with coma, the manifestations are loss of muscle tone of limbs, no spontaneous movement, disappearance of primitive reflex, most of them have increased cranial pressure and convulsion). The exclusion criteria for neonates included patients with incomplete clinical data and with congenital cardiopulmonary diseases, genetic or metabolic diseases.

### Ascertainment of variables

Background data and exposure to considered potential risk factors were extracted from the neonatal HIE database. The possible risk factors studied included neonatal birth weight, gestational age at birth, parity(one, two, three or more), feeding pattern(breastfeeding, artificial feeding or mixed feeding),delivery mode(natural delivery or cesarean section),1-min Apgar’s score (≥ 7 or < 7), 5-min Apgar’s score(≥ 7 or < 7), preterm birth, intrauterine infection, intrauterine distress, amniotic fluid contamination, amniotic fluid anomaly and abnormal labor stage. In this study, neonatal birth weight was categorized as low birth weight (1500-2499 g),normal birth weight (2500-4000 g) and macrosomia(> 4000 g). Neonatal gestational age at birth were classified as premature infant (32–36 weeks),term infant (37–42 weeks) and postterm infant (> 42 weeks). Intrauterine distress was referred to neonatal heart less than 120 beats/min or more than 160 beats/min. Amniotic fluid anomaly was defined as the amniotic fluid volume ≥ 2000 ml or ≤ 300 ml, and the amniotic fluid dark area > 7 cm or ≤ 2 cm by B-ultrasound examination. Abnormal labor stage was referred to the total labor course ≤ 3 h or ≥ 24 h, the second labor stage ≥ 2 h for primipara and ≥ 1 h for parturient. Neonatal preterm birth, intrauterine infection, intrauterine distress, amniotic fluid contamination, amniotic fluid anomaly and abnormal labor stage were all classified as a dichotomous variable “Yes” or “No”.

### Statistical analysis

Maternal and neonatal baseline descriptive characteristics with continuous variables and categorical variable were all presented as mean ± standard deviation value and frequencies and percentages respectively. Comparisons between different levels neonatal HIE were conducted with the t-test or Wilcoxon rank-sum test for continuous variables and the Chi-square test for categorical variables. Considering that there still were nonmatching variables among different levels neonatal HIE apart from age, gender, and residence region, we carried out unconditional multiple logistic regression models to estimate the independent risk factors of interest for predicting neonatal moderate or severe HIE. Collinearity between potential confounding variables was examined using Spearman rank-order correlation for continuous variables or Chi-square test for category variables. All variables identified as statistically significant (*P* < 0.05) predictors in the univariate analysis were included as candidate predictors in the multivariate model. Odds ratios(ORs) of mild HIE vs. moderate or severe HIE were reported along with the corresponding 95% confidence intervals (CIs).First, we estimated the unadjusted ORs by each maternal and neonatal characteristic in model 1. Thereafter, we adjusted ORs for the different maternal and neonatal characteristics for each other, based on associations in present data and in previous reports in model 2.

To further explore the characteristics of 228 males and females neonatal HIE in different group, we conducted trend test analysis stratified by the neonatal birth weight and gestational age at birth. In addition, we also used an additive model to test for possible biological interactions between neonatal birth weight and gestational age at birth. OR was calculated for each category after adjustment for covariates and Relative Excess Risk of Interaction (RERI) was calculated based on the additive model. All statistical analyses were conducted with IBM software SPSS (version 21, Chicago, IL, USA). All statistical tests were two-tailed and considered to be statistically significant at *P* value less than 0.05.

## Results

A total of 228 neonates recorded in the Henan Children’s Hospital Patient Register as diagnosed with neonatal HIE were included in this cross-sectional study. Of these, 21(9.2%) neonates were diagnosed with mild HIE, 207(90.8%) neonates were diagnosed with moderate or severe HIE. The demographic characteristics of mild HIE and moderate or severe HIE were summarized in Table 1. The males had a statistically significantly higher frequency compared with the females between the two groups (*P* = 0.030). However, there were no difference between the groups with respect to the residential region (*P* = 0.423). In the population,171(75.0%) neonatal birth weight were categorized as low birth weight (1500-2499 g),33(14.5%) as normal birth weight (2500-4000 g) and 24(10.5%) as macrosomia(> 4000 g). 157(67.5%) neonatal gestational age at birth were categorized as premature infant (32–36 weeks),43(18.9%) as term infant (37–42 weeks) and 31(13.6%) as postterm infant (> 42 weeks). There were statistically significantly difference between the two groups regarding neonatal birth weight and gestational age at birth(*P* < 0.05). Meanwhile, all other considered characteristics tested with significant difference between mild HIE and moderate or severe HIE(all *P* < 0.05).

**Table 1:** Maternal and neonatal demographic characteristics with different levels neonatal hypoxic ischemic encephalopathy

Variable	Total n(%)	mild HIE n(%)	moderate or severe HIE n(%)	*P*-value
Age at diagnosis(days)	13.9 ± 5.5	14.1 ± 5.7	13.7 ± 5.2	0.061
Gender				
Male	174(76.3)	12(57.1)	162(78.3)	0.030
Female	54(23.7)	9(42.9)	45(21.7)	
Residential region				
Urban	90(39.5)	10(47.6)	80(38.6)	0.423
Rural	138(60.5)	11(52.4)	127(61.4)	
Birth weight(g)				
1500–2499	171(75.0)	11(52.4)	160(77.3)	0.042
2500–4000	33(14.5)	6(28.6)	27(13.0)	
> 4000	24(10.5)	4(19.0)	20(9.7)	
Gestational age at birth (weeks)				
32–36	154(67.5)	9(42.9)	145(70.0)	0.040
37–42	43(18.9)	7(33.3)	36(17.4)	
> 42	31(13.6)	5(23.8)	26(12.6)	
Parity				
1	134(58.7)	8(38.1)	126(60.9)	0.034
2	64(28.1)	11(52.4)	53(25.6)	
≥ 3	30(13.2)	2(9.5)	28(13.5)	
Feeding pattern				
Breastfeeding	135(59.2)	9(42.9)	126(60.9)	0.035
Artificial feeding	56(24.6)	10(47.6)	46(22.2)	
Mixed feeding	37(16.2)	2(9.5)	35(16.9)	
Delivery mode				
Natural delivery	134(58.8)	8(38.1)	126(60.9)	0.043
Cesarean section	94(41.2)	13(61.9)	81(39.1)	
1-min Apgar’s score				
≥ 7	137(60.1)	7(33.3)	130(62.8)	0.009
< 7	91(39.9)	14(66.7)	77(37.2)	
5-min Apgar’s score				
≥ 7	116(50.9)	15(71.4)	101(48.8)	0.048
< 7	112(49.1)	6(28.6)	106(51.2)	
Preterm birth				
Yes	172(75.4)	11(52.4)	161(77.8)	0.010
No	56(24.6)	10(47.6)	46(22.2)	
Intrauterine infection				
Yes	98(43.0)	15(71.4)	83(40.1)	0.006
No	130(57.0)	6(28.6)	124(59.9)	
Intrauterine distress				
Yes	119(52.2)	16(76.2)	103(49.8)	0.021
No	109(47.8)	5(23.8)	104(50.2)	
Amniotic fluid contamination				
Yes	122(53.5)	16(76.2)	106(51.2)	0.029
No	106(46.5)	5(23.8)	101(48.8)	
Amniotic fluid anomaly				
Yes	130(57.0)	17(81.0)	113(54.6)	0.020
No	98(43.0)	4(19.0)	94(45.4)	
Abnormal labor stage				
Yes	172(75.4)	12(57.1)	160(77.3)	0.041
No	56(24.6)	9(42.9)	47(22.7)	

The detection and trend analysis results of mild HIE and moderate or severe HIE among neonates with different birth weight and different gestational age at birth were presented in Table 2. We found a trend association between different levels neonatal HIE and birth weight, with the decreased of the neonatal birth weight, the detection rates of mild HIE and moderate or severe HIE in males and females were gradually increased (all *P*_trend_ < 0.05). The detection rates of different levels HIE in males and females were the highest in low birth weight (1500-2499 g). The association between different levels neonatal HIE and gestational age at birth also showed a tendency, with the decreased of the neonatal gestational age at birth, the detection rates of mild HIE and moderate or severe HIE in males and females were gradually increased (all *P*_trend_ < 0.05). The detection rates of different levels HIE in males and females were the highest in premature infant (32–36 weeks).

**Table 2:** Detection and trend test analysis of different levels HIE among neonates with different groups

Group	Male			Female		
	Total	mild HIE	moderate or severe HIE	Total	mild HIE	moderate or severe HIE
Birth weight(g)						
1500–2499	135	6(50.0)	129(79.6)	36	5(55.6)	31(68.9)
2500–4000	23	5(41.7)	18(11.1)	12	3(33.3)	9(20.0)
> 4000	16	1(8.3)	15(9.3)	6	1(11.1)	5(11.1)
*P*_trend*_		0.016	0.028		0.021	0.039
Gestational age at birth(weeks)						
32–36	115	5(41.7)	110(67.9)	39	6(66.7)	33(73.3)
37–42	31	4(33.3)	27(16.7)	13	2(22.2)	11(24.4)
> 42	28	3(25.0)	25(15.4)	2	1(11.1)	1(2.3)
*P*_trend*_		0.023	0.035		0.037	0.042

Multivariate logistic regression analysis of influencing neonatal moderate or severe HIE was shown in Table 3. Compared with the normal birth weight (2500-4000 g), neonates as low birth weight (1500-2499 g) (adjusted *OR*:1.965, 95%*CI*:1.086–4.127)was independent risk factor of moderate or severe HIE after adjusting for potential confounders in model 2. Also, neonatal gestational age at birth exposed to premature infant (32–36 weeks) (adjusted *OR*:1.557, 95%*CI*:1.589–4.862) increased the risk of moderate or severe HIE compared with term infant (37–42 weeks). Table 4 showed the interaction analysis results between neonatal birth weight and gestational age at birth, the RERI and 95%CI in neonatal was 0.821(-0.046,1.687) and indicated that no interaction was detected based on the additive model. In addition, the results revealed that neonatal 1-min Apgar’s score < 7, intrauterine distress, amniotic fluid contamination were significantly associated with an increased risk of moderate or severe HIE.

**Table 3:** Independent risk factors for neonatal moderate or severe HIE based on logistic regression analysis

Variable	Model 1^a^		Model 2^b^	
	Unadjusted OR(95%CI)	*P*-value	Adjusted OR(95%CI)	*P*-value
Birth weight(g)				
1500–2499	1.701(1.153 ~ 3.082)	0.019	1.965(1.086 ~ 4.127)	0.036
2500–4000	1.00(ref)*		1.00(ref)	
> 4000	2.879(0.234 ~ 4.328)		3.212(0.597 ~ 5.161)	
Gestational age at birth(weeks)				
32–36	1.152(1.067 ~ 2.985)	0.027	1.557(1.589 ~ 4.862)	0.041
37–42	1.00(ref)		1.00(ref)	
> 42	2.983(0.719 ~ 5.106)		3.027(0.165 ~ 6.017)	
1-min Apgar’s score				
≥ 7	1.00(ref)	< 0.001	1.00(ref)	0.009
< 7	4.253(3.592 ~ 6.315)		5.618(3.724 ~ 7.353)	
Intrauterine distress				
Yes	5.921(3.565 ~ 6.828)	0.002	4.916(3.431 ~ 7.398)	0.011
No	1.00(ref)		1.00(ref)	
Amniotic fluid contamination				
Yes	2.775(1.986 ~ 4.357)	< 0.001	3.965(2.153 ~ 5.782)	0.025
No	1.00(ref)		1.00(ref)	

^a^Model 1: the unadjusted ORs by each maternal and neonatal characteristic

^b^Model 2: adjusted ORs for the different maternal and neonatal characteristics for each other

*1.00(ref) meant the reference group

**Table 4:** The RERI analysis results between neonatal birth weight and gestational age at birth bases on the additive model

Variable	Estimate	S.E	Wald	*P*-value	Lower	Upper
OR_00_	0.161	0.075	2.185	0.031	0.022	0.3045
OR_01_	0.424	0.042	11.013	< 0.01	0.356	0.508
OR_10_	0.223	0.056	4.245	< 0.01	0.122	0.332
OR_11_	0.615	0.027	26.622	< 0.01	0.571	0.665
OR_11_-OR_10_	0.392	0.065	6.864	< 0.01	0.284	0.514
OR_01_-OR_00_	0.263	0.082	3.165	< 0.01	0.102	0.426
RERI	0.821	0.447	1.876	0.066	-0.046	1.687

## Discussion

Moderate or severe HIE is one of the most common diseases in neonatal period. Therefore, understanding the onset and related risk factors affecting the neonatal moderate or severe HIE, can help clinicians take preventive measures and improve the prognosis of neonates thus reducing the mortality and disability rate in time. The development of moderate or severe HIE is caused by the interaction of multiple factors throughout the neonatal period. In this cross-sectional study, we compared the differences among various factors of neonates with moderate and severe HIE, and the results showed that five factors was associated with higher risk of neonatal moderate or severe HIE after adjusting for other variables. The adjusted logistic regression analysis showed significant associations between neonatal moderate or severe HIE and low birth weight, premature infant,1-min Apgar’s score < 7, intrauterine distress and amniotic fluid contamination. No biological interaction was detected between neonatal birth weight and gestational age at birth based on the additive model.

In the neonatal factors, our study showed that low birth weight was associated with an increased risk of moderate or severe HIE(adjusted *OR*:1.965, 95%*CI*:1.086–4.127).The trend analysis results also found that with the decreased of the neonatal birth weight, the detection rates of moderate or severe HIE in males and females were gradually increased (all *P*_trend_ < 0.05), and the detection rates of moderate or severe HIE in males and females were the highest in low birth weight. The relationship between neonatal premature infant and moderate or severe HIE also showed the same results. Our finding was consistent with the previous literature, in a retrospective study of 17,706 newborns, Futrakul et al. revealed that gestational age were significant risk factors of HIE [24]. Neonatal low birth weight and premature infant were always accompanied by imperfect organ development, especially immature lung development, which was easily lead to the occurrence of moderate or severe HIE. However, no biological interaction between neonatal birth weight and gestational age at birth based on the additive model was found in this study, that meant when multiple risk factors interact with moderate or severe HIE, the effect was equivalent to the sum of the independent effects of the two factors.

It has been suggested that the Apgar’s score is a feasible and practical indicator to evaluate the degree of neonatal moderate or severe HIE, especially the 1-min Apgar’s score is still a valid and rapid index to assess the effectiveness of resuscitative efforts and the presence and degree of respiratory depression in neonates [25]. Our study indicated that there was a statistically significant relationship between 1-min Apgar’s score < 7 and moderate or severe HIE(adjusted *OR*:5.618, 95%*CI*:3.724–7.353). In addition, our data reported that intrauterine distress was also associated with a significant increase in risk of neonatal moderate or severe HIE (adjusted *OR*:4.916, 95%*CI*:3.431–7.398). The present study supported the previous report by Wang J^[[[26]]]^, that Apgar’s score (*OR*:5.648, 95%*CI*:3.704–9.241) and intrauterine distress(*OR*:3.662, 95%*CI*:2.085–4.736) were independent risk factors for HIE. Several studies have demonstrated that the continuous lower Apgar’s score indicated the higher degree of neonatal moderate or severe HIE, which can caused hypoxia and acidosis, redistributing blood, significant abnormalities of residual alkali,blood glucose and failure of brain energy metabolism, resulting in brain edema and cell damage, or death in severe neonates [27,28]. Hypoxia caused by low Apgar’s score is also a continuation result of intrauterine distress in utero mostly. Intrauterine distress is the manifestation of acute or chronic fetal hypoxia. Long-term hypoxia can cause acidosis, inhibit respiratory center and lead to neonatal moderate or severe HIE. Therefore, these results remind that it is necessary to carry out the 1-min Apgar’s score and disease degree assessment in time to help clinicians to take targeted treatment measures and strengthen the monitoring of the whole labor process, thus avoid the occurrence of asphyxia and reduce the brain damage caused by asphyxia in neonatal moderate or severe HIE.

Amniotic fluid contamination was also a risk factor in this study and has received a lot of attention. Our study concluded that after adjusting for potential confounders in multiple logistic regression models, neonates with amniotic fluid contamination were 3.965 times more likely to develop moderate or severe HIE than those without amniotic fluid contamination(adjusted *OR*:3.965, 95%*CI*:2.153–5.782). Our finding was consistent with the previous literature, Wang J et al. carried out a retrospective case–control study in southern China, in which they found that *OR* for neonatal HIE associated with amniotic fluid contamination was 4.527(95%*CI*:2.704–5.483) (using 306 cases, 306 controls) [19]. Some studies have suggested that neonate exposed to amniotic fluid contamination is an important factor leading to intrauterine distress, and there is likely to be a positive association between amniotic fluid contamination and neonatal moderate or severe HIE[29,30]. The higher degree of amniotic fluid contamination, the higher probability of neonates with moderate or severe HIE. Neonates with intrauterine hypoxia, hyperperistalsis, anal sphincter relaxation, can make meconium discharge into the amniotic fluid. Currently amniotic fluid contamination is a clinical manifestation of neonatal intrauterine hypoxia, the incidence of neonatal HIE increases when amniotic fluid contamination occurs. Therefore, prenatal examination should be used to monitor whether the amniotic fluid is contaminated. Once the neonate is in distress, correct countermeasures should be made to end the delivery with appropriate methods timely.

The main strengthens of the present study are the enrolled population of neonates came from a single hospital institution and adjustment for various covariates in the analysis, thus minimizing differences in outcome assessment and limiting information bias. But several limitations of the study should be noted. Firstly, our study mainly focused on the neonatal moderate or severe HIE, such restrictions lead to a reduction in the sample size of our study, which may have caused some variables with lack of significance. Secondly, given that the study was conducted in a cross-sectional design, the causal pathways underlying the observed relationships could hardly be verified. Further cohort studies are needed if permitted. The selected controls in this study were recruited among neonates who were diagnosed with mild HIE, some factors which limited to data collected from patients’ medical records could still differ between cases and controls, leading to unrecognized selection bias. It is very hard to balance all these baseline factors in the process of recruiting cases and controls. Thirdly, utilizing diagnosis codes to identify cases which may caused small number of cases cannot be screened out with this method, some of the risk estimates were based on small numbers and some might be statistically significant simply by chance alone, this may have impacted our ability to establish an association between neonatal moderate or severe HIE and other factors. Our finding need to be replicated by other investigations in the future with lager sample sizes. Finally, in our neonatal HIE database we lacked of the information of HIE therapy and when we designed the study before, we failed to investigate and collect the relevant treatment data. Therefore, the relevant in-depth study including detection, diagnosis and therapy of neonatal HIE roundly is necessary in the future, in order to carry out early screening and epidemiological monitoring of moderate or severe HIE patients to achieve the "three early" goals of early detection, early diagnosis and early intervention.

## Conclusions

In conclusion, findings in our study are in line with the previous literatures, which show that neonates with low birth weight, premature infant,1-min Apgar’s score < 7, intrauterine distress, amniotic fluid contamination are risk factors for moderate or severe HIE. Notably, we found no biological interaction between risk factors based on the additive model. It is important to pay close attention to these risk factors, which may help to inform prevention and early detection strategies, as this may succeed in minimizing the incidence of neonatal moderate or severe HIE.

## Data Availability

The datasets used and/or analysed during the current study are available from the corresponding author on reasonable request.

## Abbreviations

ORs: Odds Ratios
CIs: Confidence Intervals
RERI: Relative Excess Risk of Interaction
